# Assessment of myocardial metabolic disorder associated with mediastinal radiotherapy for esophageal cancer -a pilot study-

**DOI:** 10.1186/s13014-015-0410-z

**Published:** 2015-04-21

**Authors:** Rei Umezawa, Kentaro Takanami, Noriyuki Kadoya, Yujiro Nakajima, Masahide Saito, Hideki Ota, Haruo Matsushita, Toshiyuki Sugawara, Masaki Kubozono, Takaya Yamamoto, Yojiro Ishikawa, Ken Takeda, Yasuyuki Taki, Kei Takase, Keiichi Jingu

**Affiliations:** Department of Radiation Oncology, Tohoku University Graduate School of Medicine, Sendai, Japan; Department of Diagnostic Radiology, Tohoku University Graduate School of Medicine, Sendai, Japan; Department of Radiological Technology, School of Health Sciences, Faculty of Medicine, Tohoku University, Sendai, Japan; Department of Developmental Cognitive Neuroscience Institute of Development, Aging and Cancer, Tohoku University, Sendai, Japan

**Keywords:** Radiotherapy, Radiation-induced myocardial damage, I-123 BMIPP, Esophageal cancer, Heart

## Abstract

**Background:**

To evaluate the dose-effect relations for myocardial metabolic disorders after mediastinal radiotherapy (RT) by performing iodine-123 β-methyl-iodophenyl pentadecanoic acid (I-123 BMIPP) scintigraphy.

**Methods:**

Between 2011 and 2012, we performed I-123 BMIPP scintigraphy for patients with esophageal cancer before and six months after curative mediastinal RT. Single photon emission computed tomography (SPECT) images of pre-RT and post-RT were registered into RT dose distributions. The myocardium was contoured, and the regional RT dose was calculated. Normalization is required to compare pre- and post-RT SPECT images because the uptake pattern is changed due to the breathing level. Normalization was applied on the mean of SPECT counts in regions of the myocardium receiving less than 5 Gy. Relative values in each dose region (interval of 5 Gy) were calculated on the basis of this normalization for each patient. The reduction in the percent of relative values was calculated.

**Results:**

Five patients were enrolled in this study. None of the patients had a past history of cardiac disease. The left ventricle was partially involved in RT fields in all patients. The patients received RT with median total doses of 60-66 Gy for the primary tumor and metastatic lymph nodes. Concomitant chemotherapy consisting of cisplatin or nedaplatin and 5-fluorouracil with RT was performed in 4 patients. All patients had reduced uptake corresponding to RT fields. Dose-effect relations for reduced uptake tended to be observed at 6 months after RT with mean decreases of 8.96% in regions at 10-15 Gy, 12.6% in regions at 20-25 Gy, 15.6% in regions at 30-35 Gy, 19.0% in regions at 40-45 Gy and 16.0% in regions at 50-55 Gy.

**Conclusions:**

Dose-effect relations for myocardial metabolic disorders tended to be observed. We may need to make an effort to reduce high-dose mediastinal RT to the myocardium in RT planning.

## Background

Several studies have revealed that long-term survivors with Hodgkin’s lymphoma, breast cancer and esophageal cancer after mediastinal radiotherapy (RT) have RT-induced heart disease (RIHD) such as pericarditis, myocardial ischemia, cardiomyopathy, heart failure, valvular abnormalities, or conduction defects [1-5]. RIHD is one of the late complications that radiation oncologists must be aware of. One of the pathological mechanisms of RIHD is microvascular injury that occurs within several months of RT [6]. Irradiation to the myocardium causes damage to the microvasculature, leading to inflammatory and thrombotic changes, capillary loss, focal ischemia, and interstitial fibrosis [6,7]. In some past studies, myocardial perfusion defects corresponding to RT fields were found by myocardial perfusion scintigraphy [8,9].

The onset of myocardial ischemia is followed by metabolic disorder, left ventricular dysfunction, electrocardiographic changes and angina [10]. The main energy source of the myocardium changes from free fatty acids to glucose and lactate under ischemic conditions [11]. In our previous study, we reported that reduced uptake (myocardial metabolic disorder) corresponding to RT fields was observed mainly in the base of the left ventricle by using scintigraphy with iodine-123 β-methyl-iodophenyl pentadecanoic acid (I-123 BMIPP), which is a branched fatty acid analogue that enters myocardial cells and can show the degree of myocardial fatty acid metabolism [12]. However, we did not perform I-123 BMIPP scintigraphy before RT as a limitation of that study.

The purpose of the present study was to evaluate myocardial metabolic disorders corresponding to RT fields by performing I-123 BMIPP scintigraphy before and after mediastinal RT. We also evaluated the dose-effect relations for reduced uptake. We report these results as a pilot study.

## Methods

### Patients

Between 2011 and 2012, we performed I-123 BMIPP scintigraphy for patients with early stage esophageal cancer before and six months after curative mediastinal RT. This study was approved by a local institutional review board, and all of the patients gave written informed consent before enrolment.

### I-123 BMIPP scintigraphy (myocardial fatty acid imaging)

Single photon emission computed tomography (SPECT) imaging was performed 20 minutes after intravenous administration of 111 MBq of I-123 BMIPP (Cardiodine® Injectable, provided by Nihon Mediphysics Co, Sendai, Japan), using a dual-detector gamma camera combined with computed tomography (CT) (Symvia T2, Siemens, Hoffman Estates, IL). The SPECT scan was performed with a low-energy, high-resolution collimator, a matrix of 64 × 64 (pixel size, 3.3 × 3.3 mm) and an acquisition time of ninety 25-s frames over 360°. The 159 keV photopeak for I-123 with a 24% window was selected for data acquisition. No downscatter correction was performed. The SPECT images were reconstructed iteratively using 3-dimensional ordered-subsets expectation maximization with 8 iterations and 5 subsets with a 3D spatial Gaussian filter (9.6 mm in full width at half maximum). We performed SPECT/CT in the condition of expiration to compare with the RT fields more easily and to detect anatomical locations more clearly.

### Radiotherapy

Gross tumor volume was defined as the primary tumor and nodal metastasis based on a CT scan and positron emission tomography-CT. If it was difficult to discriminate the primary tumor on the RT planning CT, the clips were placed on the proximal and distal sides of the primary tumor. Initial clinical target volume (CTV) was defined as the region from the supraclavicular to celiac lymph nodes. Initial CTV was made small in consideration of the patient’s general condition. Boost CTV was defined as the primary tumor with a 20-30-mm craniocaudal margin and an approximately 5-mm radial margin and nodal metastasis. Planning target volume was defined as CTV plus a 5-15-mm margin. The initial CTV received 40 Gy using parallel-opposed anterior-posterior fields. The boost CTV received 20-26 Gy using parallel-oblique fields to avoid the spinal cord. The total dose was 60-66 Gy. Treatment planning was performed by CT in all patients, and the dose distribution was determined by ECLIPSE Varian Medical Systems (Palo Alto CA) with the analytical anisotropic algorithm. The dose volume histogram (DVH) of the myocardium was calculated. By reference to the literature of Feng et al. [13], the myocardium was contoured. The inner cavity of the left ventricle was excluded.

### Deformable image registration (DIR)

We performed CT separately at the planning for initial CTV and boost CTV. Basically, although we can add them with the same planning CT image, it is impossible to add two dose distributions with different planning CT images. The deformable image registration (DIR) technique enables us to make dose accumulation with dose warping [14-18]. Using dose warping, we created total dose distribution by adding the initial and boost plans. In this study, DIR with the region of interest (ROI) technique implemented in a 3D slicer (Brigham and Women’s Hospital, Boston, MA) was performed. The DIR algorithm was B-spline. This algorithm has already been validated [18]. First, DIR was performed between initial planning CT (reference image) and boost planning CT (moving image) to create a transformation using the ROI. The ROI was basically defined to cover the dose distribution. Then resultant transformation was applied to the boost dose distribution to create the deformed boost dose distribution, which was a warped dose distribution according to the reference CT image. The accuracy was judged by the quality of alignment from visual inspection. Finally, we added the two dose distributions to create total dose distribution.

### Registering SPECT images with RT dose distribution

To quantify post-RT myocardial metabolic disorder in the myocardium, all SPECT images at pre- and post-RT and a planning CT image with dose distribution needed to be defined on the same coordinate space. Automatic rigid registration with the ROI technique was performed. The steps consisted of (a) the ROI being basically defined to cover the heart, (b) automatic rigid registration performed using the ROI to align the two CT images (reference: planning CT image, moving: pre- or post-RT CT image with perfusion) and judgment of the quality of alignment from visual inspection, (c) manual registration performed if adjustment needed and (d) the derived translations and rotations applied to pre- or post-RT SPECT perfusion.

### Evaluation of the relations between RT dose and myocardial metabolic disorder

**Figure 1:**
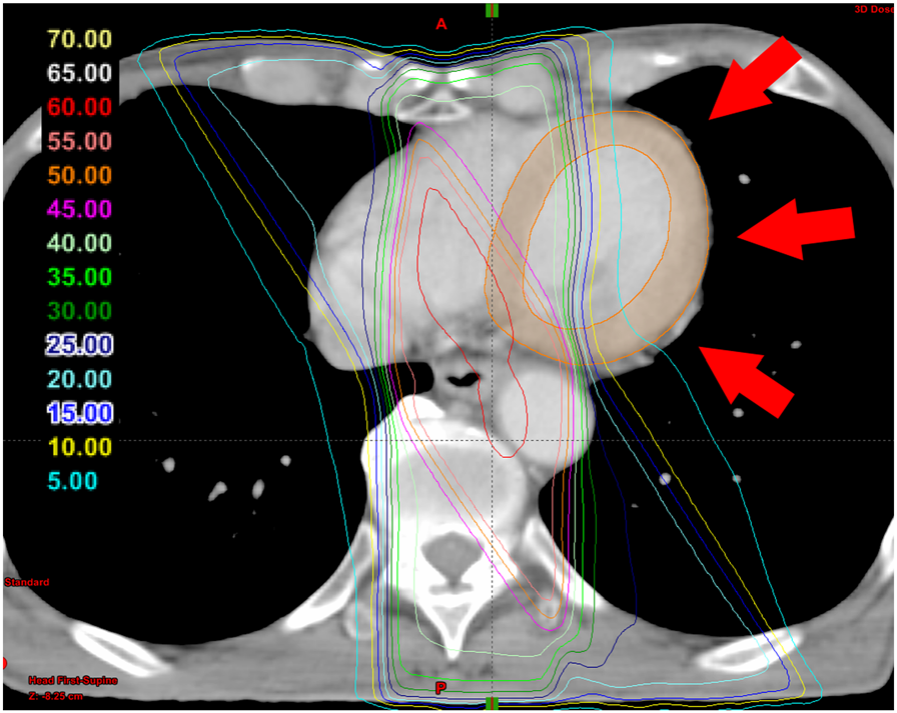
Evaluation of the relations between radiation dose and myocardial metabolic disorder. Normalization was applied on the mean of single photon emission computed tomography counts in regions of the myocardium receiving less than 5 Gy (red arrow). Relative values in each dose region (interval of 5 Gy) were calculated on the basis of this normalization for each patient. The reduction in the percent of relative values was calculated.

Normalization is required to compare pre- and post-RT SPECT images because the uptake pattern is changed due to the breathing level and the circumstance of hunger before I-123 BMIPP scintigraphy. By reference to the report by Seppenwoolde et al. [19], normalization was applied on the mean of SPECT counts in the regions of the myocardium receiving less than 5 Gy, assuming that RT-induced microvascular injury is not present at this low dose (Figure 1). The mean of SPECT counts in each dose region (interval of 5 Gy) was determined, and relative values in each dose region were calculated on the basis of this normalization for each patient. This process was performed at the I-123 BMIPP examinations of pre- and post-RT. The reduction in the percent of relative values at pre- and post-RT SPECT was calculated in each dose region.

### Cardiac examination

Changes in brain natriuretic peptide (BNP) level, electrocardiogram (ECG) and pericardial effusion were determined after RT in all patients. We investigated whether reduced uptake reflected those changes.

## Results

**Figure 2:**
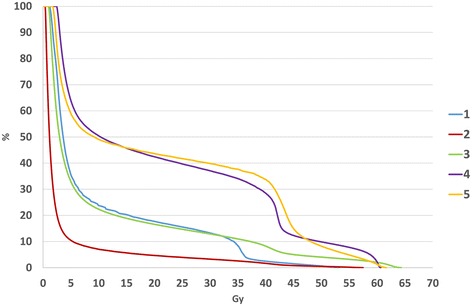
Dose volume histograms of the myocardium for 5 patients.

Five patients were enrolled in this study. None of the patients had a past history of cardiac disease. The left ventricle was partially involved in RT fields in all patients. Patients’ characteristics are shown in Table 1. Concomitant chemotherapy consisting of 2 cycles of cisplatin or nedaplatin and 5-fluorouracil (5-FU) with RT was performed in 4 patients. DVHs of the myocardium for 5 patients are shown in Figure 2. The means of V10, V20, V30, V40 and V50 of the myocardium were about 30.6%, 25.0%, 21.2%, 14.9% and 4.65%, respectively. V30 of the myocardium in two patients (Case 4 and Case 5) was more than 30%.

**Table 1:** **Patient characteristics**

**Case**	**Age**	**Sex**	**TNM**	**Primary site**	**Smoking**	**HT**	**DM**	**HL**	**Chemotherapy**	**RT dose**
1	75	Male	T1bN0M0	Lt	+	-	-	-	CDGP + 5-FU	60 Gy/30 fr
2	82	Male	T1bN0M0	Lt	+	+	-	-	CDGP + 5-FU	60 Gy/30 fr
3	82	Male	T1bN0M0	Mt	+	-	-	+	None	66 Gy/33 fr
4	73	Male	T3N0M0	Mt	+	-	-	-	CDDP + 5-FU	60 Gy/30 fr
5	73	Male	T1bN0M0	Lt	+	-	-	-	CDDP + 5-FU	60 Gy/30 fr

*Abbreviations*: *HT* hypertension, *DM* diabetes, *HL* hyperlipidemia, *RT* radiation, *Lt* lower thoracic esophagus, *Mt* middle thoracic esophagus, *CDGP* Nedaplatin, *CDDP* cisplatin, *5-FU* 5-fluorouracil.

**Figure 3:**
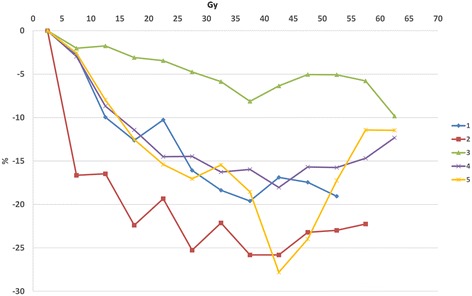
Results of dose-effect relations for myocardial metabolic disorder. Dose-effect relations for reduced uptake tended to be observed.

**Figure 4:**
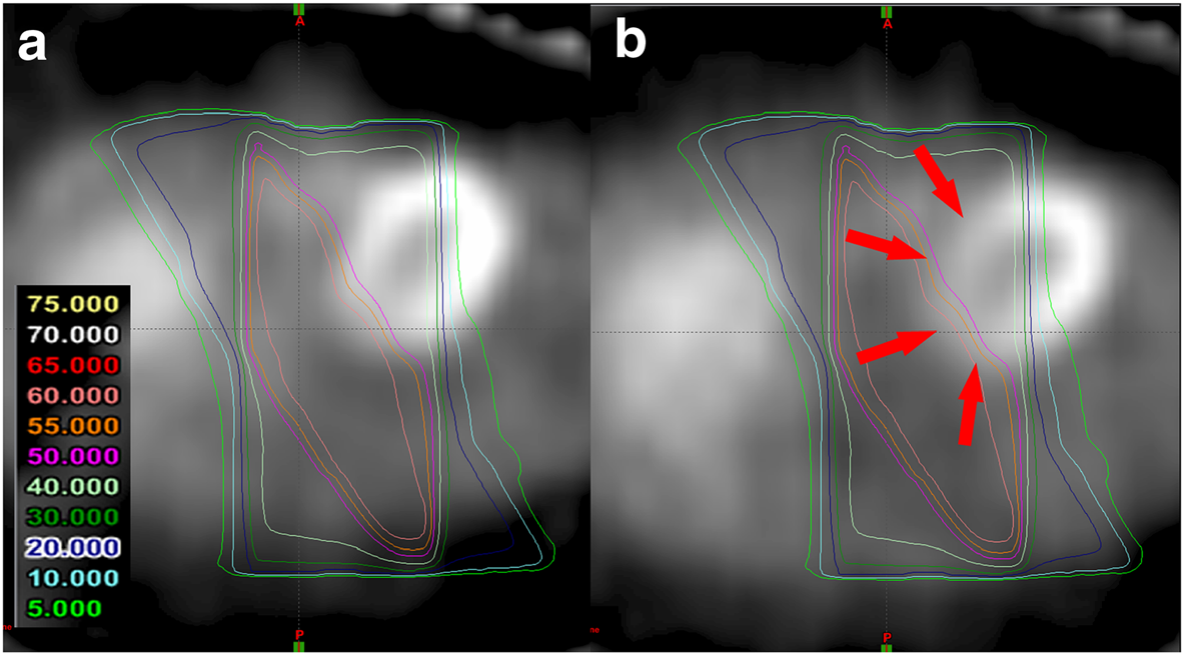
Findings of single photon emission computed tomography (SPECT) in Case 5. Reduced uptake corresponding to anterior-posterior fields was observed (red arrow). **(a)** pre-RT SPECT. **(b)** post-RT SPECT.

All patients had reduced uptake corresponding to RT fields. The results of dose-effect relations for myocardial metabolic disorder are shown in Figure 3. Dose-effect relations for reduced uptake tended to be observed at 6 months after RT with mean decreases of 8.96% in regions at 10-15 Gy, 12.6% in regions at 20-25 Gy, 15.6% in regions at 30-35 Gy, 19.0% in regions at 40-45 Gy and 16.0% in regions at 50-55 Gy. From visual inspection, reduced uptake corresponding to RT fields was distinct in Case 4 and Case 5. Images of Case 5 are shown in Figure 4. In this case, reduced uptake corresponding to anterior-posterior fields (40 Gy line) was observed.

The results of cardiac examinations are shown in Table 2. None of the patients had symptoms when they underwent I-123 BMIPP scintigraphy. BNP level increased after RT in four patients, and mean levels of BNP at pre-RT and post-RT were 26.32 pg/ml and 58.44 pg/ml, respectively. One patient had ECG change after RT (from no abnormality to T wave abnormality). Three patients had pericardial effusion after RT.

**Table 2:** **Cardiac examinations**

**Case**	**ECG (pre-RT)**	**ECG (post-RT)**	**BNP (pre-RT)**	**BNP (post-RT)**	**Pericardial effusion**
1	Left axis deviation	Left axis deviation	41.5 pg/ml	65.1 pg/ml	+
2	Abnormal PR interval	Normal	11.0 pg/ml	20.4 pg/ml	-
3	Normal	Normal	46.9 pg/ml	149.7 pg/ml	-
4	Normal	Normal	24.9 pg/ml	18.8 pg/ml	+
5	Normal	T wave abnormality	7.3 pg/ml	38.2 pg/ml	+

*Abbreviations*: *ECG* electrocardiogram, *RT* radiation, *BNP* brain natriuretic peptide.

## Discussion

In the present study, reduced uptake (myocardial metabolic disorders) corresponding to RT fields was observed as it was in our previous study. Although I-123 BMIPP scintigraphy has not been used routinely compared with myocardial perfusion scintigraphy, some studies showed that I-123 BMIPP is superior to perfusion imaging for evaluating the extent and severity of damage to the myocardium in patients with acute and old myocardial infarctions [20,21]. Moreover, I-123 BMIPP scintigraphy can be performed at rest and side effects of I-123 BMIPP scintigraphy are rare. Therefore, we consider I-123 BMIPP scintigraphy to be useful examination for detecting RT-induced myocardial damage.

Although reductions of fatty acid metabolism occurred six months after RT, indicating the possibility of RT-induced microvascular injury, Marks et al. also reported that RT-induced myocardial perfusion defects were detected six months after RT for breast cancer [8]. However, the association between early myocardial metabolic disorders and clinically important events in the future has been unclear. All of the patients in the present study were asymptomatic when BMIPP scintigraphy was performed. However, elevation of BNP levels in four patients and ECG change in one patient were documented, though the association between myocardial metabolic disorders and those cardiac examinations has also not been clear. Previously, we also reported that there was a significant difference between BNP values in patients without abnormal 18F-fluorodeoxyglucose (FDG) accumulation in the irradiated myocardium and those in patients with abnormal FDG accumulation after RT for esophageal cancer [22]. Based on those points, we need to take careful note of these changes to check the presence or absence of RIHD. Because Case 5 in which reduced uptake corresponding to RT fields was distinct had elevation of BNP levels, ECG change and pericardial effusion, we intend to follow up this patient more carefully.

Dose-effect relations for reduced uptake rate were also observed. Hardenberg et al. evaluated dose-effect regional cardiac perfusion abnormalities in patients with left-sided breast cancer treated with RT [23]. They reported that a dose-effect perfusion defect was seen at 6 months with minimal defect at 0-10 Gy, 7% decrease in regional perfusion at 11-20 Gy, 11% decrease in regional perfusion at 21-30 Gy, 15% decrease in regional perfusion at 41-50 Gy and 20% decrease in regional perfusion at 41-50 Gy. The pattern of dose-effect abnormalities in their study was similar to that in our study. In an experimental study by Seemann et al., mouse hearts were irradiated with a dose of 2, 8, or 16 Gy, and structural and functional changes were monitored to shed light on the dose dependence of the severity and rate of progression of cardiovascular damage [24]. They reported that microvascular density in the left ventricle was significantly decreased at 40 weeks after 16 Gy and that alkaline phosphatase was significantly decreased at 40 weeks after 2, 8 and 16 Gy. At 40 weeks, half of the hearts irradiated with 2 Gy and almost all of the hearts irradiated with 8 or 16 Gy showed vascular leakage. Dose-effect microcascular injury was also detected by pathological examinations.

In the present study, high-dose irradiation to the myocardium induced strong myocardial metabolic disorder. Gayed et al. found that most of the myocardial perfusion defects were encompassed within isodose lines of more than 45 Gy using myocardial perfusion scintigraphy for esophageal cancer [9]. In studies by Mulrooney et al. and Schellong et al., cardiac RT dose of > 35 Gy increased the incidence of cardiac disease [1,25]. One of the risk factors of RIHD has been reported to be a high cumulative dose of RT (>30 Gy) [26]. From visual inspection, reduced uptake corresponding to RT fields was distinct in Case 4 and Case 5. V30 of the myocardium in those cases was more than 30%. Marks et al. reported that RT-induced myocardial perfusion defects were correlated with the volume of irradiated myocardium (receiving greater than 50% of prescribed dose) in patients who received a total dose of 46 to 50 Gy for left-sided breast cancer [8]. Based on the results of those clinical studies, we may need to make an effort to reduce high-dose RT (>30 Gy) to the myocardium in RT planning. Because esophageal cancer and lung cancer primary are often next to heart and require larger doses for curative treatment, we need to perform RT planning more carefully. A four-field technique with the combination of parallel-opposed anterior-posterior and parallel-oblique fields may be useful to reduce the incidence of RT-induced cardiac disease, especially in middle and lower thoracic esophageal cancer.

In the present study, low-dose irradiation to the myocardium also induced mild myocardial metabolic disorder. The correlation between low-dose irradiation to the heart and clinical events is not clearly known. Tukenova et al. evaluated cardiovascular mortality after childhood cancer [27]. They reported that the risk of cardiac disease was significantly higher in individuals who received an average RT dose exceeding 5 Gy (relative risk, 12.5 and 25.1 for 5 to 14.9 Gy and > 15 Gy, respectively). Mulrooney et al. reported that cardiac RT dose of > 15 Gy increased the relative hazards of congestive heart failure, myocardial infarction, pericardial disease and valvular abnormalities by twofold to sixfold compared to non-RT survivors among adult survivors of childhood and adolescent cancers [25]. Considering that mild microvascular injury was also detected in the study by Seemann et al. [24], intensity-modulated radiotherapy is one of the solutions to reduce high-dose irradiation to the myocardium, but we might also need to pay careful attention to the possibility of a cardiac effect of low-dose mediastinal RT.

Reduced uptake was milder in Case 3 than in the other cases in the present study. Case 3 received RT alone. Although accurate assessment was difficult due to the small number of cases in the present study, concomitant chemotherapy might increase reduced uptake. Chemotherapy with a platinum-based agents (cisplatin or nedaplatin) and 5-FU was used in the present study. Some studies showed that cisplatin was associated with cardiotoxicity [28,29]. Cisplatin-based chemotherapy is known to be associated with increased production of reactive oxygen species, leading to damage of the myocardium [28]. Barjaktarovic et al. demonstrated that the level of reactive oxygen species was enhanced in high-dose irradiated cardiac mitochondria [30], and increased reactive oxygen species by interaction between platinum-based agents might have affected myocardial metabolic disorders in the present study.

There are some limitations in the present study. First, the number of cases was too small to analyze our results accurately. We have since been obtaining results from more cases. Second, there were some difficulties in registering SPECT images with RT dose distribution because of the difference in respiratory conditions for SPECT images at pre- and post-RT and planning CT. CT scans were performed in the conditions of expiration for the I-123 BMIPP examination and free breathing for the RT planning. The regions at 50-55 Gy existed mainly in the base of the myocardium. The base of the myocardium was susceptive to change in reduced uptake due to the condition of respiration in the present study, and there is the possibility of the degree of reduced uptake having been underestimated or overestimated. Therefore, the degree of reduced uptake in regions at 50-55 Gy might have been less than that in regions at 40-45 Gy in the present study.

## Conclusions

Dose-effect relations for reduced uptake tended to be observed. We may need to make an effort to reduce high-dose mediastinal RT (>30 Gy) to the myocardium in RT planning as much as possible. We will analyze more cases and analyze dose-effect myocardial metabolic disorders in more detail using a larger number of cases.

## Abbreviations

RT: Radiotherapy
I-123 BMIPP: Iodine-123 β-methyl-iodophenyl pentadecanoic acid
SPECT: Single photon emission computed tomography
RIHD: RT-induced heart disease
CT: Computed tomography
CTV: Clinical target volume
DVH: Dose volume histogram
DIR: Deformable image registration
ROI: Region of interest
BNP: Brain natriuretic peptide
ECG: Electrocardiogram
5-FU: 5-fluorouracil
FDG: 18F-fluorodeoxyglucose
